# Humoral Immunogenicity of mRNA-1010, an mRNA-based Seasonal Influenza Vaccine, in Adults ≥50 Years

**DOI:** 10.1093/ofid/ofag646

**Published:** 2026-10-13

**Authors:** Isabel Leroux-Roels, Elissa Malkin, HuiLing Chen, Andrei Avanesov, Ren Chen, Brianna Fidler, Rituparna Das, Eleanor Wilson

**Affiliations:** Department of Diagnostic Sciences, Center for Vaccinology, Ghent University and Ghent University Hospital, Ghent, Belgium; Moderna, Inc., Cambridge, Massachusetts, USA; Moderna, Inc., Cambridge, Massachusetts, USA; Moderna, Inc., Cambridge, Massachusetts, USA; Moderna, Inc., Cambridge, Massachusetts, USA; Moderna, Inc., Cambridge, Massachusetts, USA; Moderna, Inc., Cambridge, Massachusetts, USA; Moderna, Inc., Cambridge, Massachusetts, USA

**Keywords:** hemagglutination inhibition, immunogenicity, mRNA influenza vaccine, seasonal influenza vaccine

## Abstract

**Background:**

Seasonal influenza causes substantial morbidity and mortality worldwide. In a phase 3 study in adults ≥50 years, trivalent mRNA-1010 demonstrated superior efficacy vs a standard-dose (SD) seasonal influenza vaccine. This analysis reports humoral immunogenicity.

**Methods:**

Participants were randomized 1:1 to a single dose of mRNA-1010 (37.5-µg) or licensed SD influenza vaccine. Immunogenicity was assessed in a prespecified per-protocol subset. Antihemagglutinin antibody titers were measured by hemagglutination inhibition (HAI) and microneutralization (MN; subset) at baseline and day 29 against vaccine-matched strains. Outcomes included geometric mean titers (GMTs), geometric mean fold rises (GMFRs) from baseline, and seroconversion rates (SCRs; ≥4-fold rise).

**Results:**

The Per-Protocol Immunogenicity Subset included 2342 participants (mRNA-1010, n = 1167; SD influenza vaccine, n = 1175). mRNA-1010 induced higher levels of HAI antibodies relative to SD influenza vaccine. mRNA-1010 increased HAI GMTs, with day 29 GMFRs of 4.07 (A/H1N1), 4.70 (A/H3N2), and 3.14 (B/Victoria). Geometric mean ratio point estimates vs SD influenza vaccine exceeded 1.5 across all strains, with lower bounds (LBs) of 95% CIs all exceeding 1.4: 1.82 (95% confidence interval [CI], 1.69–1.97), 1.59 (1.48–1.71), and 1.67 (1.57–1.78), respectively. Similarly, SCR differences were positive; LBs >15: 27.95% (95% CI: 24.09–31.73), 19.99% (15.99–23.92), and 25.33% (21.65–28.95), respectively. mRNA-1010 elicited higher MN antibody responses vs SD influenza vaccine for all 3 strains.

**Conclusions:**

In adults ≥50 years, a single dose of mRNA-1010 induced higher humoral immune responses against vaccine-matched influenza strains than licensed SD influenza vaccine. Together with previously reported results, these findings support mRNA-1010 as a seasonal influenza vaccine option in adults ≥50 years.

**Clinical Trials Registration:**

NCT06602024.

## Teaser

A single dose of trivalent mRNA-1010 elicited robust hemagglutination inhibition antibody responses against A/H1N1, A/H3N2, and B/Victoria in adults ≥50 years, demonstrating superior immunogenicity vs a standard-dose seasonal influenza vaccine.

Seasonal influenza remains a major cause of global morbidity and mortality, with an estimated 3–5 million cases of severe illness and up to 650 000 deaths annually worldwide [1]. Adults aged ≥50 years bear a disproportionate burden of influenza-related disease, including a higher risk of complications, hospitalizations, and deaths, reflecting age-related declines in immune function and a higher prevalence of chronic comorbidities [2-4]. As a result, influenza prevention in this population remains a public health priority. There are multiple licensed options available for the older adult population, but there remains room for improvement. Interim mid-season estimates of the effectiveness of licensed influenza vaccines for the 2024–2025 influenza season in the United States ranged from 18% to 57% in adults aged ≥65 years across care settings [5]. In older adults, age-associated changes in immune responsiveness further influence vaccine-induced protection and have contributed to the development of enhanced vaccine formulations, including high-dose, recombinant protein, and adjuvanted vaccines [6, 7].

Influenza vaccines primarily target the hemagglutinin (HA) surface glycoprotein, a key viral antigen involved in receptor-binding and membrane fusion and the principal mediator of virus neutralization. Although neuraminidase also contributes to protective immunity, currently licensed influenza vaccines are standardized based on HA antigen quantity [8]. Antibody responses to HA are largely strain-specific, and antigenic drift in HA each season contributes to variability in vaccine effectiveness [9, 10]. Hemagglutination inhibition (HAI) titers, which reflect functional HA-specific antibodies, are well-established immunologic biomarkers for influenza vaccines and are commonly used as surrogate measures of protection [9]. Messenger RNA (mRNA)–based vaccine platforms represent an alternative approach for seasonal influenza vaccination, enabling flexible antigen design and rapid adaptation in response to updated strain recommendations, avoiding egg-based manufacturing-associated antigenic changes, and potentially eliciting more robust or durable immune responses in older adults compared with traditional formulations [11].

mRNA-1010 is an investigational trivalent seasonal influenza vaccine that encodes the surface HA antigens corresponding to the World Health Organization (WHO)'s recommended strains for a given season (12.5 µg per strain). Clinical evaluation of mRNA-1010 has shown that the vaccine can generate a robust immune response in adults. In a phase 1/2 study, mRNA-1010 demonstrated broad humoral and cellular immune responses against influenza A and B and elicited broader antibody response against heterologous influenza strains compared with a licensed standard-dose (SD) comparator [12, 13]. mRNA-1010 was further optimized by introducing amino acid substitutions to influenza B HA to improve expression while preserving antigenic sites and enhancing immune responses against influenza B strains [14]. In a subsequent phase 3 study, mRNA-1010 induced HAI titers against vaccine-matched influenza A and B strains that were superior to those elicited by a licensed SD influenza vaccine (Fluarix® Quadrivalent [SD-IIV4]; GlaxoSmithKline Biologicals, Dresden, Germany) in adults 18–64 years and by a licensed high-dose (HD) influenza vaccine (Fluzone® HD Quadrivalent [HD-IIV4]; Sanofi Pasteur Inc., Swiftwater, PA, United States) in adults aged ≥65 years [15]. In a separate randomized phase 1 study, mRNA-1010 elicited humoral and cellular immune responses that were overall comparable to those induced by the MF59-adjuvanted influenza vaccine FLUAD® (Seqirus Inc.; Holly Springs, NC, United States), including similar HAI responses across strains and evidence of enhanced antigen-specific memory B-cell and T-cell responses [16]. Data from these studies demonstrate that mRNA-1010 induces a broad immune response, one that can be comparable to or higher than immune responses demonstrated by licensed high-dose or MF59-adjuvanted vaccines.

This phase 3 study (FLUENT) was conducted during the 2024–2025 Northern Hemisphere influenza season to evaluate the efficacy, safety, and immunogenicity of mRNA-1010 compared with a licensed inactivated SD influenza vaccine in adults aged ≥50 years. In this trial, a single dose of mRNA-1010 demonstrated superior relative vaccine efficacy of 26.6% (95% confidence interval [CI], 16.7–35.4) against influenza illness compared with the SD comparator, with no safety concerns identified [17]. The current report describes the day 29 postvaccination humoral immunogenicity findings from this study.

## METHODS

### Study Design and Participants

This phase 3, randomized, double-blind, active-controlled trial (NCT06602024) evaluated the safety, efficacy, and immunogenicity of mRNA-1010 in adults aged ≥50 years; additional study design details are provided in the Supplementary Data. This report presents humoral immunogenicity results from a subset of approximately 2400 participants enrolled at sites in North America. Hemagglutination inhibition antibody responses were assessed against the 3 vaccine-matched strains recommended by the WHO for the 2024–2025 Northern Hemisphere season for cell culture–, recombinant protein– or nucleic acid–based vaccines: Influenza A H1N1/Wisconsin/67/2022, Influenza A H3N2/Massachusetts/18/2022, and Influenza B Victoria/Connecticut/01/2021. Microneutralization (MN) assays assessed neutralizing antibody (nAb) responses in approximately 500 participants from this subset. Immunogenicity analyses were conducted at 2 time points, day 1 (prevaccination baseline) and day 29 postvaccination; additional methodological details are provided in the Supplementary Data.

Eligible participants were medically stable adults aged ≥50 years. Key exclusion criteria included clinically unstable conditions that could interfere with safety, immune response, or adherence to study procedures, as well as receipt of a seasonal influenza vaccine within 6 months prior to study vaccination. Full eligibility criteria are provided in the Supplementary Data.

### Patient Consent Statement

All participants provided written informed consent prior to enrollment. The study was conducted in accordance with the International Council for Harmonisation Good Clinical Practice guidelines and the principles of the Declaration of Helsinki. The protocol was approved by all applicable institutional review boards.

### Objectives

The immunogenicity objective of this phase 3 trial was to evaluate humoral immune responses to trivalent mRNA-1010 relative to a licensed SD influenza vaccine, an active comparator, against vaccine-matched influenza A/H1N1, A/H3N2, and B/Victoria strains (see “Statistical Analysis” for further details). Endpoints included HAI geometric mean titers (GMTs) and seroconversion rates (SCRs) on day 29 (defined as either a baseline HAI titer <1:10 and a postinjection titer ≥1:40 or a baseline HAI titer ≥1:10 and a minimum 4-fold rise in postinjection HAI titer) and geometric mean fold rise (GMFR). The GMFR was calculated as the ratio of the day 29 to the day 1 (baseline) GMT within each vaccine group. Additional endpoints included the percentage of participants achieving an HAI titer ≥1:40 at day 29 and between-group differences in SCRs.

An exploratory objective was to evaluate nAb responses measured by MN assay in a prespecified subset of participants from the immunogenicity cohort. Microneutralization endpoints included GMTs at day 29, GMFRs from baseline, and correlations between MN and HAI responses.

### Study Interventions and Procedures

Participants were randomly assigned (1:1) to receive trivalent mRNA-1010 (37.5 μg total; 12.5 μg per strain) or a licensed egg-based SD influenza vaccine comparator. Licensed SD trivalent vaccines were used where available; in countries where trivalent formulations were not available, licensed quadrivalent SD vaccines were administered. Comparator vaccines were Fluarix®, Fluarix® Tetra, Influsplit® Tetra, and Alpharix® Tetra (GlaxoSmithKline Biologicals, Dresden, Germany), inactivated egg-based SD influenza vaccines containing WHO-recommended strains for the 2024‒2025 Northern Hemisphere season. These comparators were selected based on their broad availability across participating regions and to provide consistency across geographies. Study vaccines were administered as a single intramuscular injection into the deltoid muscle.

Serum samples for immunogenicity assessments were collected on day 1 (prevaccination) and day 29 from all participants. Of these, samples from a prespecified random subset of participants were analyzed for immunogenicity endpoints. Hemagglutination inhibition antibody titers were measured using validated assays at IQVIA Laboratories (Durham, NC, United States). Neutralizing antibody responses were evaluated using a validated MN assay performed at VisMederi (Siena, Italy). All sample testing was conducted in a blinded manner. Detailed assay procedures are provided in the Supplementary Data.

### Statistical Analysis

The Immunogenicity Subset was a prespecified subset of all randomized participants who received any study vaccination and had day 1 (baseline) and day 29 humoral immunogenicity samples and were analyzed according to the study vaccination group to which they were randomized. The Per-Protocol Immunogenicity Subset (PPIS) for HAI assay analyses consisted of participants from the Immunogenicity Subset who received the assigned study vaccination, had evaluable day 1 (baseline) and day 29 immunogenicity samples, and had no important protocol deviations that may have impacted the immunogenicity assessment. The PPIS included approximately 2400 participants, selected using a stratified random sampling approach and enrolled at North American sites where an SD trivalent influenza vaccine was used as the comparator. Participants with reverse transcriptase–polymerase chain reaction–confirmed influenza infection between days 1 and 29 were excluded from the PPIS to support interpretation of the association of vaccine-induced antibody titers and study outcomes. Among participants randomly selected for the HAI assay, an additional randomization was done to select approximately 500 participants for the MN assay to ensure that participants selected for the MN assay also had HAI results.

Immunogenicity analyses were descriptive. Hemagglutination inhibition antibody responses were summarized by study group for each vaccine-matched strain (A/H1N1, A/H3N2, and B/Victoria). Geometric mean titers with corresponding 95% CIs were calculated at baseline and day 29. The 95% CIs were calculated based on the t-distribution of the log-transformed values and then back-transformed to the original scale. Geometric mean fold rises of HAI titers at day 29 over day 1 (baseline) were summarized with corresponding 95% CIs.

An analysis of covariance (ANCOVA) model was used to estimate the GMT of each arm and the geometric mean ratio (GMR) to assess the differences in immune response between mRNA-1010 vs SD influenza vaccine at day 29. Immunogenicity analyses were descriptive, with no formal hypothesis testing planned. A lower bound of the 95% CI for the GMR >0.667 was used as a benchmark for interpretation; additional details are provided in the Supplementary Methods. The number and percentage of participants who achieved seroconversion at Day 29 were provided, with 2-sided 95% CIs calculated using the Clopper–Pearson method. Differences in SCRs between study intervention groups were estimated using the Miettinen–Nurminen method to calculate 95% CIs for each strain. In addition, the number and percentage of participants with an HAI titer ≥1:40 at day 29 were summarized with 2-sided 95% CIs using the Clopper–Pearson method.

Neutralizing antibody responses measured by MN assay were analyzed using the same statistical approach. Microneutralization endpoints included GMTs at baseline and Day 29 and GMFRs from baseline. Microneutralization GMTs and between-group comparisons were analyzed using the same ANCOVA model as for the HAI analysis.

The GMR and SCR differences for influenza A and B strains at day 29 were assessed by subgroups of age, sex, race, body mass index (BMI), prior year influenza vaccine status (yes or no), and high-risk status. High-risk participants were defined as those with a BMI ≥30 kg/m^2^, a medical history of chronic conditions, including autoimmune or immune-mediated disorders; hematologic, cardiac, pulmonary, renal, hepatic, nervous system, or mental health disorders; or diabetes mellitus.

Statistical analyses were performed using SAS software, version 9.4 (SAS Institute; Cary, NC, United States).

## RESULTS

### Participants

A total of 40 805 participants were enrolled in the FLUENT study between 16 September 2024 and 7 March 2025. A subset of 2394 participants were included in the immunogenicity subset to assess humoral immune responses. Of these, 2342 participants were included in the PPIS (1167 in the mRNA-1010 group and 1175 in the SD influenza vaccine group), which served as the population for the immunogenicity analyses presented below. A total of 52 participants (2.2%) were excluded from the PPIS, most commonly because of noncompliance with the timing of immunogenicity blood sampling (26 participants; 1.1%) (Figure 1).

**Figure 1.**
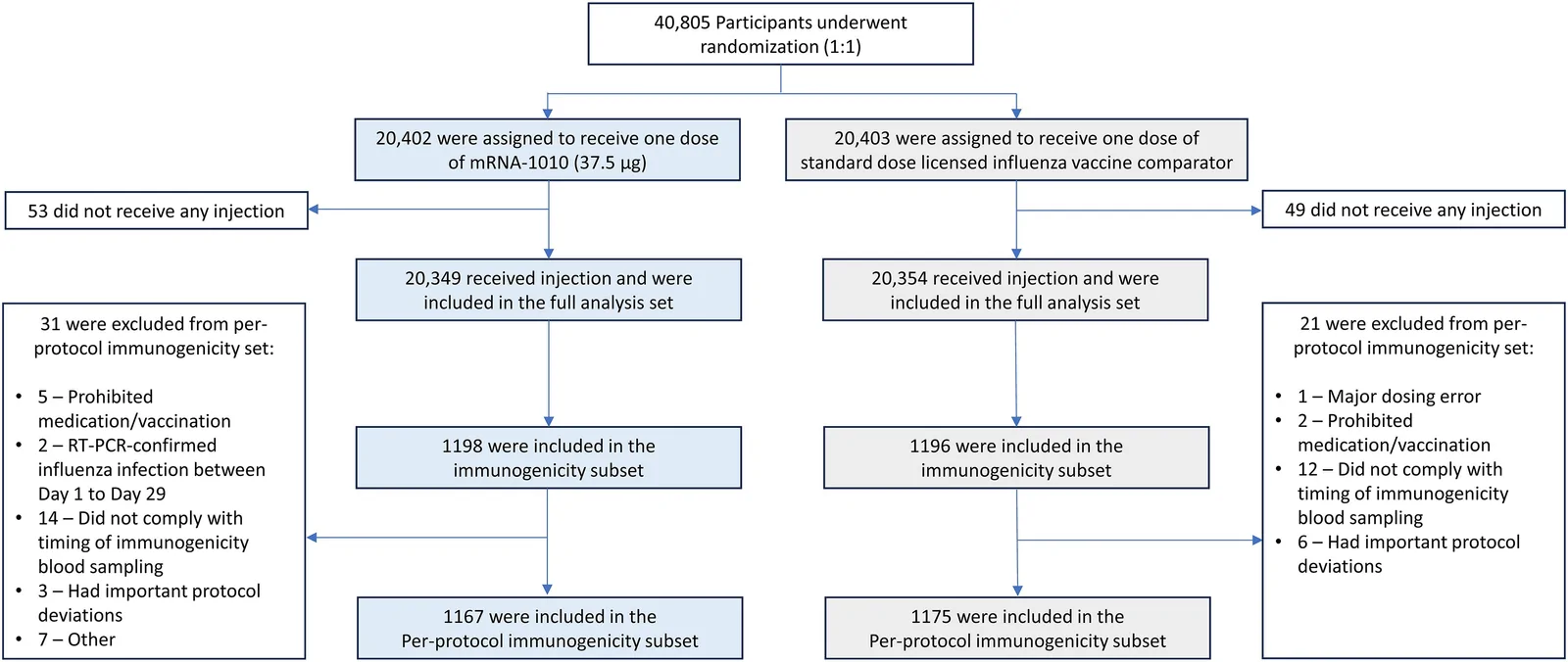
Participant flow diagram. The full analysis set (FAS) includes all participants who received any study vaccine. The immunogenicity subset includes participants in the FAS who received a standard-dose influenza vaccine or mRNA-1010 and have baseline and day 29 antibody data. The Per-Protocol Immunogenicity Subset includes participants in the immunogenicity subset and without any important protocol deviations that could adversely affect the immunogenicity assessment. The immunogenicity subset includes participants who were selected using a stratified random sampling approach and enrolled at North American sites where a standard-dose trivalent influenza vaccine was used as the comparator. “Other” exclusion reasons include protocol deviations that would have risked data biases and participants who enrolled into the study multiple times. Abbreviations: RT-PCR, reverse transcription polymerase chain reaction.

Baseline demographics and clinical characteristics in the PPIS were well balanced between the mRNA-1010 and SD influenza vaccine groups and were similar to the full study population. The median (range) age of participants was 64.0 (50–97) years: 58.4% were female, 77.9% were White, and 14.0% identified as Hispanic or Latino (Table 1). More than half (50.8%) had received a prior seasonal influenza vaccine. Overall, 63.7% of participants had ≥1 baseline high-risk factor. The Edmonton Frail Scale was assessed in participants aged ≥65 years; among those with an evaluable score, 24.8% were classified as vulnerable or frail (Edmonton Frail Scale score ≥4) [18].

**Table 1.** Participant Demographics and Characteristics in the Per-Protocol Immunogenicity Subset

Characteristic	mRNA-1010 (N = 1167)	Standard-Dose Licensed Influenza Vaccine (N = 1175)	Total (N = 2342)
Age at enrollment, y			
Mean (standard deviation)	64.6 (8.42)	64.5 (8.43)	64.5 (8.43)
Median (min, max)	65.0 (50, 97)	64.0 (50, 92)	64.0 (50, 97)
Age group, no. (%)			
≥50 to <65 y	581 (49.8)	592 (50.4)	1173 (50.1)
≥65 to <75 y	437 (37.4)	434 (36.9)	871 (37.2)
Sex, no. (%)			
Male	485 (41.6)	490 (41.7)	975 (41.6)
Female	682 (58.4)	685 (58.3)	1367 (58.4)
Race, no. (%)			
White	914 (78.3)	910 (77.4)	1824 (77.9)
Black	193 (16.5)	200 (17.0)	393 (16.8)
Asian	27 (2.3)	33 (2.8)	60 (2.6)
Other^a^	20 (1.7)	21 (1.8)	41 (1.8)
Unknown	2 (0.2)	2 (0.2)	4 (0.2)
Not reported	11 (0.9)	9 (0.8)	20 (0.9)
Ethnicity, no. (%)			
Hispanic or Latino	163 (14.0)	165 (14.0)	328 (14.0)
Not Hispanic or Latino	982 (84.1)	991 (84.3)	1973 (84.2)
Not reported	19 (1.6)	18 (1.5)	37 (1.6)
Unknown	3 (0.3)	1 (<0.1)	4 (0.2)
BMI (kg/m^2^)			
n	1165	1175	2340
Mean (standard deviation)	30.38 (6.882)	30.34 (6.855)	30.36 (6.867)
Median (min, max)	29.50 (15.0, 75.3)	29.40 (16.3, 65.7)	29.40 (15.0, 75.3)
Receipt of seasonal influenza vaccine in the previous season, no. (%)			
Received	594 (50.9)	596 (50.7)	1190 (50.8)
Not received	573 (49.1)	579 (49.3)	1152 (49.2)
Frailty status, no. (%)^b^			
Participants with a score, n	585	581	1166
Fit	437 (74.6)	440 (75.5)	877 (75.0)
Vulnerable	106 (18.1)	93 (16.0)	199 (17.0)
Frail	42 (7.2)	48 (8.2)	90 (7.7)
Vulnerable or frail	148 (25.3)	141 (24.3)	289 (24.8)
Missing data	1 (0.2)	2 (0.3)	3 (0.3)
Baseline high-risk status^c^			
Yes	740 (63.4)	753 (64.1)	1493 (63.7)
No	427 (36.6)	422 (35.9)	849 (36.3)

BMI = (body weight in kilograms)/(height in meters)^2^. Numbers are based on planned vaccination group, and percentages are based on the number of participants in the Per-Protocol Immunogenicity Subset.

Abbreviations: BMI, body mass index; EFS, Edmonton Frail Scale; Max, maximum; Min, minimum.

^a^Other race includes American Indian or Alaska Native, Native Hawaiian or other Pacific Islander, other, or multiple.

^b^EFS total score is only applicable to participants aged ≥65 years old, and the percentages are based on the number of participants aged ≥65 years old in the Per-Protocol Immunogenicity Subset. Fit = a score of 0–3; vulnerable = a score of 4–5; frail = a score ≥6.

^c^High-risk factors include baseline BMI ≥30 kg/m^2^, having a medical history of autoimmune/immune-mediated disease, blood disorders, cardiac disorders, diabetes mellitus, hepatic disorders, mental impairment disorders, nervous system disorders, pulmonary disorders, or renal disorders.

### Immunogenicity

#### Hemagglutination Inhibition Assay Results

Baseline GMTs were similar between the study groups for all 3 influenza strains. From baseline to day 29, GMTs increased for each strain in both groups. In the mRNA-1010 group, GMFRs (95% CI) from baseline to day 29 were 4.07 (3.84–4.31) for A/H1N1, 4.70 (4.44–4.97) for A/H3N2, and 3.14 (2.99–3.31) for B/Victoria. In the SD influenza vaccine group, GMFRs (95% CI) were 2.24 (2.11–2.37), 2.98 (2.81–3.16), and 1.88 (1.80–1.97), respectively (Figure 2). In the mRNA-1010 group, Day 29 SCRs (95% CI) were 55.0% (52.11–57.89) for A/H1N1, 60.8% (57.97–63.65) for A/H3N2, and 45.1% (42.19–47.98) for B/Victoria. In the SD influenza vaccine group, day 29 SCRs (95% CI) were 27.1% (24.54–29.70) for A/H1N1, 40.9% (38.02–43.72) for A/H3N2, and 19.7% (17.50–22.14) for B/Victoria. Across all 3 strains, day 29 SCRs and GMFRs were numerically higher in the mRNA-1010 group than in the SD influenza vaccine group.

**Figure 2.**
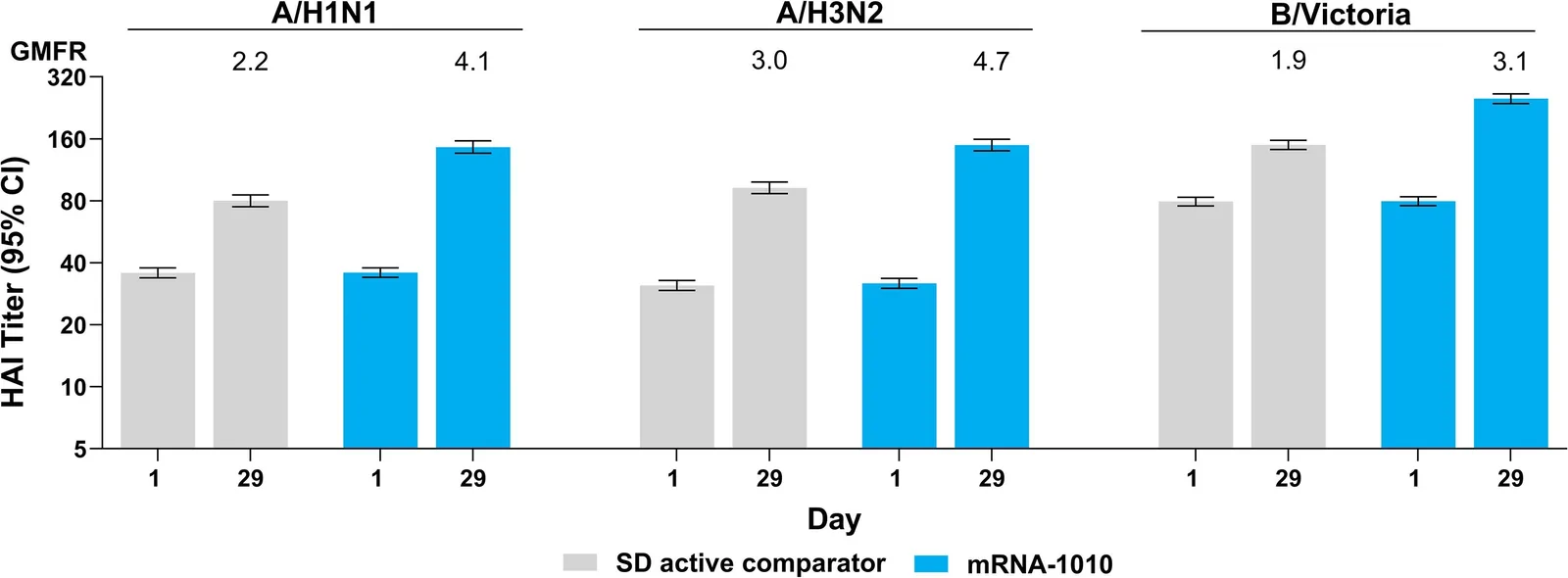
HAI GMTs and GMFRs for mRNA-1010 and SD influenza vaccines through day 29. GMFRs at day 29 from day 1 (baseline) are shown above each day 29 bar plot. Abbreviations: GMFR, geometric mean fold rise; GMT, geometric mean titer; HAI, hemagglutination inhibition; SD, standard dose.

For all 3 influenza strains, postvaccination antibody responses were higher in the mRNA-1010 group than in the SD influenza vaccine group. Day 29 GMRs (95% CIs) were 1.82 (1.69–1.97) for A/H1N1, 1.59 (1.48–1.71) for A/H3N2, and 1.67 (1.57–1.78) for B/Victoria; the lower bounds (LBs) of the 95% CIs exceeded the 0.667 benchmark for all 3 strains (Figure 3*A*). For all 3 strains, the LBs of the 95% CIs for SCR differences exceeded 15% (Figure 3*B*). Day 29 SCR differences (95% CI) were 27.95% (24.09–31.73) for A/H1N1, 19.99% (15.99–23.92) for A/H3N2, and 25.33% (21.65–28.95) for B/Victoria.

**Figure 3.**
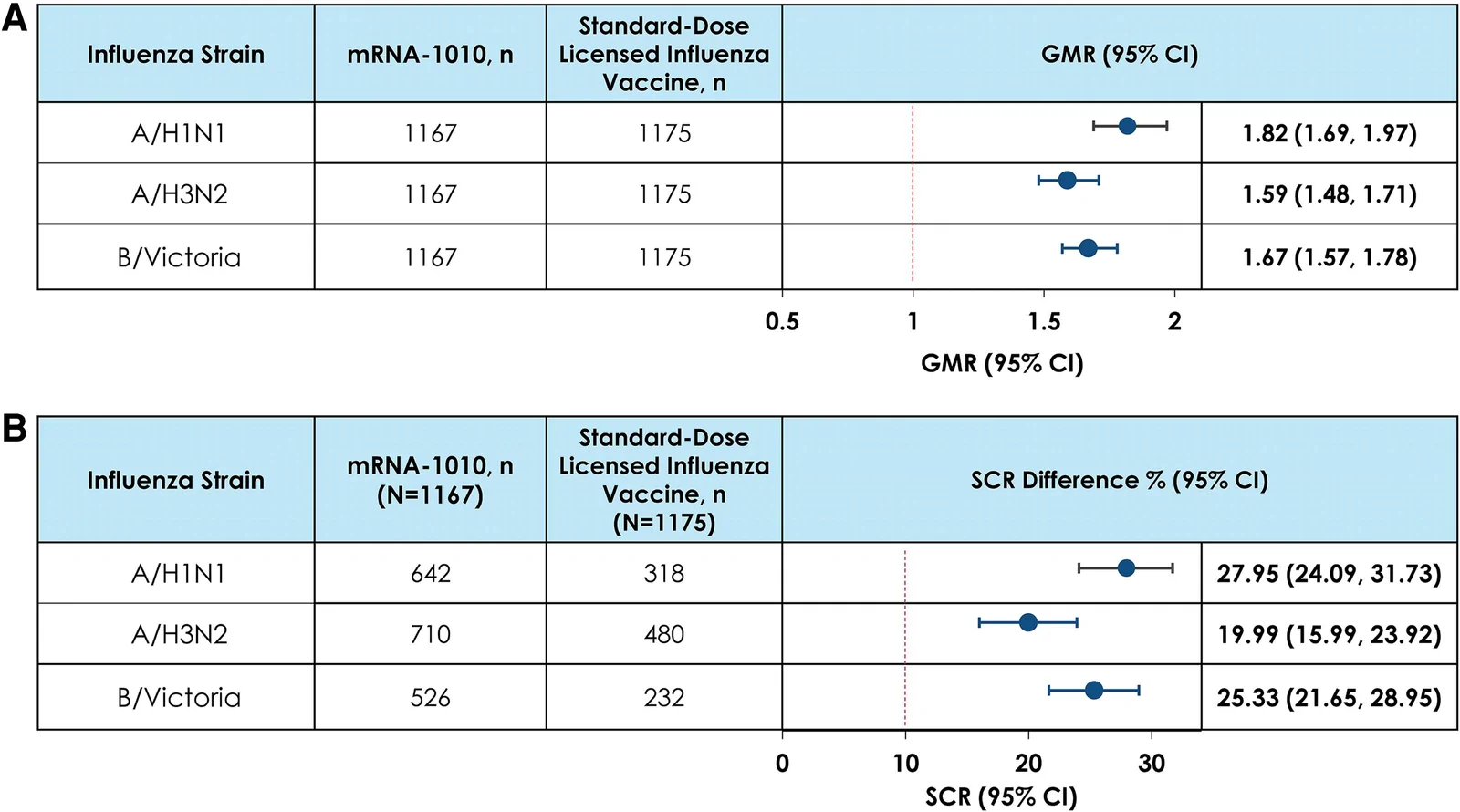
(*A*), GMR and (*B*) SCR differences in hemagglutination inhibition antibodies for mRNA-1010 vs SD influenza vaccine through day 29. Antibody values reported as below the LLOQ were replaced by 0.5 × LLOQ. Values greater than the ULOQ were converted to the ULOQ. Antibody values are shown for A/H1N1 (LLOQ: 10; ULOQ: 3620), A/H3N2 (LLOQ: 10; ULOQ: 2560), and B/Victoria (LLOQ: 10; ULOQ: 1356). Seroconversion was defined as the proportion of participants having either a postvaccination titer ≥1:40 if baseline HAI titer was <1:10, or at least a 4-fold rise in postbaseline HAI antibody titer if baseline HAI titer was ≥1:10. Error bars represent 95% CIs. Dashed lines indicate GMR = 1 (*A*) and SCR difference = 10% (*B*). Abbreviations: CI, confidence interval; GMR, geometric mean titer ratio; HAI, hemagglutination inhibition; LLOQ, lower limit of quantification; SCR, seroconversion rate; SD, standard dose; ULOQ, upper limit of quantification.

At day 29, the percentage of participants achieving an HAI titer ≥1:40 was higher in both groups, with higher percentages observed in the mRNA-1010 group vs the SD influenza vaccine group for all 3 influenza strains (A/H1N1: 92.5% vs 80.0%; A/H3N2: 93.4% vs 85.8%; B/Victoria: 99.3% vs 98.0%) (Table 2).

**Table 2.** Summary of HAI Titers ≥1:40, GMTs, Seroconversion, and Seroconversion Difference for Vaccine-Matched Seasonal Influenza A and B Strains Measured by HAI Assay by Timepoint (Per-Protocol Immunogenicity Subset)

	A/H1N1	A/H3N2	B/Victoria
Baseline (day 1), n^a^			
mRNA-1010 37.5 μg	1167	1167	1167
Titer ≥1:40, n (%)^b^	651 (55.8)	571 (48.9)	1060 (90.8)
GMT (95% CI)^c^	35.91 (34.07, 37.86)	31.83 (30.12, 33.65)	79.79 (75.92, 83.85)
Standard-dose licensed influenza vaccine	1175	1175	1175
Titer ≥1:40, n (%)^b^	645 (54.9)	568 (48.3)	1060 (90.2)
GMT (95% CI)^c^	35.80 (33.86, 37.86)	31.06 (29.37, 32.84)	79.46 (75.70, 83.41)
Day 29, n^d^			
mRNA-1010 37.5 μg	1167	1167	1167
Seroconversion^e^, n (%)^f^	642 (55.0)	710 (60.8)	526 (45.1)
95% CI^g^	(52.11, 57.89)	(57.97, 63.65)	(42.19, 47.98)
Difference (%) (mRNA-1010 vs active comparator) (95% CI)^h^	27.95 (24.09, 31.73)	19.99 (15.99, 23.92)	25.33 (21.65, 28.95)
Titer ≥1:40, n (%)^f^	1079 (92.5)	1090 (93.4)	1159 (99.3)
95% CI^g^	(90.79, 93.91)	(91.82, 94.76)	(98.65, 99.70)
Difference (%) (mRNA-1010 vs active comparator) (95% CI)^h^	12.46 (9.73, 15.23)	7.61 (5.18, 10.11)	1.36 (0.44, 2.41)
Standard-dose licensed influenza vaccine	1175	1175	1175
Seroconversion^e^, n (%)^f^	318 (27.1)	480 (40.9)	232 (19.7)
95% CI^g^	(24.54, 29.70)	(38.02, 43.72)	(17.50, 22.14)
Titer ≥1:40, n (%)^f^	940 (80.0)	1008 (85.8)	1151 (98.0)
95% CI^g^	(77.60, 82.25)	(83.66, 87.73)	(96.98, 98.69)

Antibody values reported as below the LLOQ were replaced by 0.5 × LLOQ. Values greater than the ULOQ were converted to the ULOQ.

Abbreviations: ANCOVA, analysis of covariance; CI, confidence interval; GLSM, geometric least squares mean; GM, geometric mean; GMFR, geometric mean fold rise; GMR, geometric mean ratio, estimated by the ratio of GLSM; GMT, geometric mean titer; HAI, hemagglutination inhibition; LLOQ, lower limit of quantification; Model-based GMT, model-based geometric mean titer, estimated by GLSM; N1, number of participants with nonmissing HAI antibody data at baseline (day 1) and the corresponding visit; PPIS, per-Protocol Immunogenicity Subset; SCR, seroconversion rate; ULOQ, upper limit of quantification.

^a^Number of participants with nonmissing baseline (day 1) HAI antibody data.

^b^Percentage is based on n^a^.

^c^95% CI was calculated based on the t-distribution of the log-transformed values or the difference in the log-transformed values for GM titer and GM fold rise, respectively, and then back-transformed to the original scale for presentation.

^d^Number of participants with nonmissing HAI antibody data at the corresponding visit.

^e^Rate of seroconversion is defined as the proportion of participants with either a Baseline HAI titer <1:10 and a postbaseline titer ≥1:40 or a baseline HAI titer ≥1:10 and a minimum 4-fold rise in postbaseline HAI antibody titer.

^f^Number of participants meeting the criterion at the corresponding visit. Percentage is based on N1.

^g^95% CI was calculated using the Clopper–Pearson method.

^h^95% CI was calculated using the Miettinen–Nurminen (score) method.

Geometric mean ratio and SCR differences at day 29 were assessed across prespecified subgroups defined by age, sex, race, BMI, prior season influenza vaccination status, and baseline high-risk status. Higher responses with mRNA-1010 compared with the SD influenza vaccine were evident across all subgroups. Among participants with high-risk factors, GMRs (95% CI) were 1.89 (1.71–2.08) for A/H1N1, 1.63 (1.48–1.79) for A/H3N2, and 1.71 (1.58–1.84) for B/Victoria (Figure 4*A*). Participants aged ≥65 years also demonstrated higher antibody responses with mRNA-1010 across all strains, with GMRs of 1.85 (1.66–2.05), 1.65 (1.49–1.83), and 1.75 (1.60–1.90) for A/H1N1, A/H3N2, and B/Victoria, respectively (Figure 4*B*). Participants who received an influenza vaccine in the prior season showed similar patterns, with GMRs (95% CI) of 1.94 (1.78–2.11) for A/H1N1, 1.60 (1.46–1.75) for A/H3N2, and 1.74 (1.62–1.87) for B/Victoria (Figure 4*C*). Across all 3 strains, day 29 GMRs and SCR differences observed in these subgroups were consistent with the overall results, including among adults aged ≥65 years.

**Figure 4.**
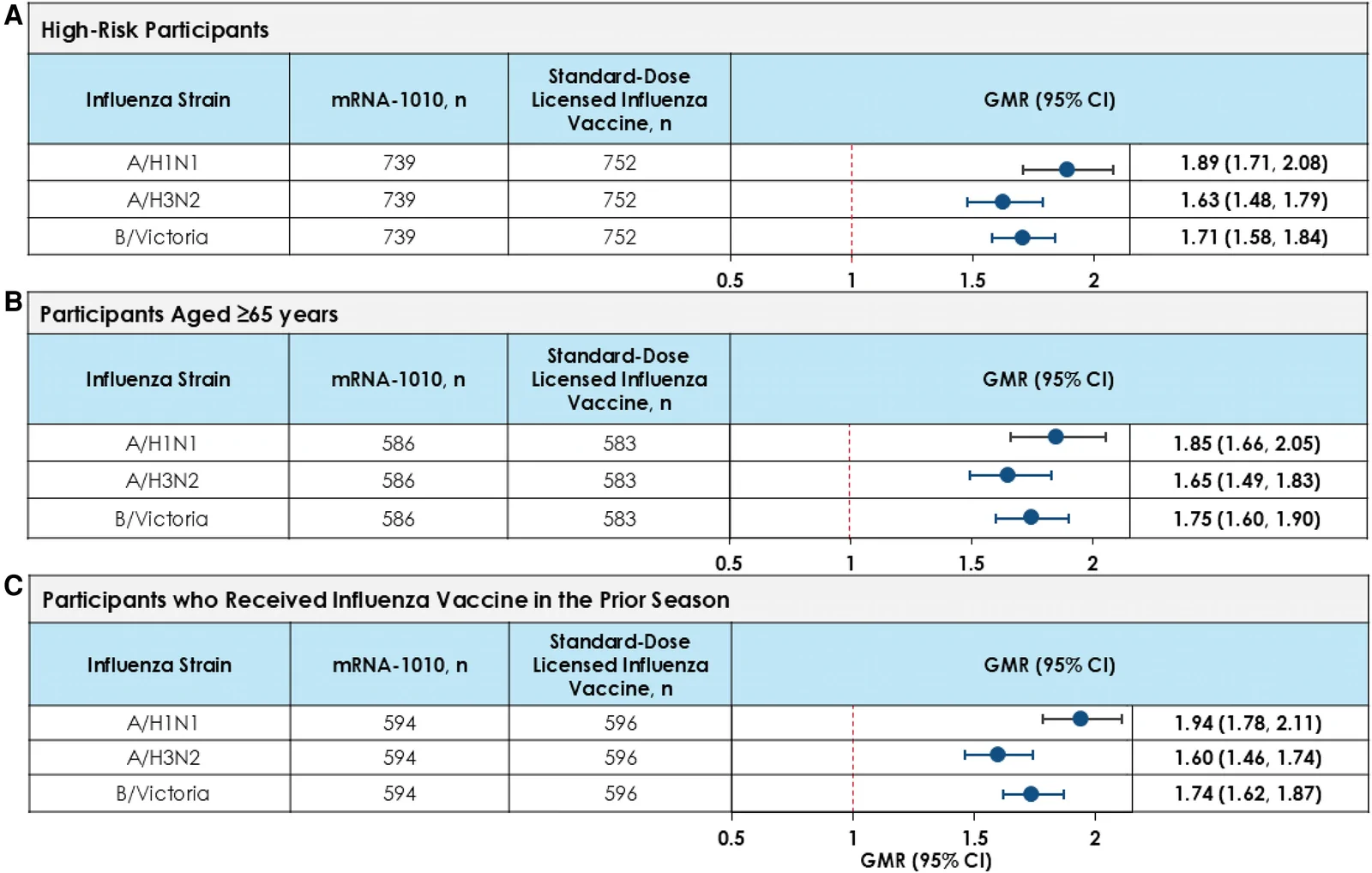
GMR of hemagglutination inhibition antibodies for mRNA-1010 vs SD influenza vaccine through day 29 in (*A*) high-risk participants and (*B*) participants aged ≥65 y and (*C*) participants who received influenza vaccine in the prior season. Antibody values reported as below the LLOQ were replaced by 0.5 × LLOQ. Values greater than the ULOQ were converted to the ULOQ. Antibody values are shown for A/H1N1 (LLOQ: 10; ULOQ: 3620), A/H3N2 (LLOQ: 10; ULOQ: 2560), and B/Victoria (LLOQ: 10; ULOQ: 1356). Error bars represent 95% CIs. Dashed lines indicate GMR = 1. High-risk factors include baseline BMI ≥30 kg/m^2^, having a medical history of autoimmune/immune-mediated disease, blood disorders, cardiac disorders, diabetes mellitus, hepatic disorders, mental impairment disorders, nervous system disorders, pulmonary disorders, or renal disorders. Abbreviations: CI, confidence interval; GMR, geometric mean ratio; LLOQ, lower limit of quantification; SD, standard dose; ULOQ, upper limit of quantification; y, years.

#### Microneutralization Assay Results

Neutralizing antibody responses were assessed by MN assay in a subset of the PPIS (mRNA-1010, n = 247; SD influenza vaccine, n = 253). Baseline MN GMTs were similar between study groups across all 3 influenza strains. At day 29, MN GMTs were higher in the mRNA-1010 group than in the SD influenza vaccine group for all 3 strains (Table 3). Day 29 GMRs for mRNA-1010 vs comparator ranged from 1.37 to 2.81, with the LBs of the 95% CIs exceeding 1.0 for all strains, indicating higher nAb responses in the mRNA-1010 group.

**Table 3.** Summary of nAb Levels (GMT) and GMR at Baseline and at Day 29 for Vaccine-Matched Seasonal Influenza A and B Strains (Per-Protocol Immunogenicity Subset Microneutralization Subset)

	mRNA-1010 37.5 μg N = 247	Standard-Dose Licensed Influenza Vaccine N = 253
Antibody: influenza A/H1N1 Wisconsin/67/2022 nAb (titer) by MN assay (LLOQ: 17, ULOQ: 9738)		
Baseline (day 1), n^a^	247	253
GMT (95% CI)^b^	56.48 (46.78, 68.19)	59.30 (49.10, 71.62)
Day 29, n^c^	247	253
GMT (95% CI)^b^	514.42 (419.43, 630.92)	190.89 (153.51, 237.35)
Model-based GMT (95% CI)	527.19 (447.53, 621.03)	187.67 (159.63, 220.63)
GMR (mRNA-1010 vs active comparator) (95% CI)	2.809 (2.231, 3.537)	
Antibody: influenza A/H3N2 Massachusetts/18/2022 nAb (titer) by MN assay (LLOQ: 12, ULOQ: 2269)		
Baseline (day 1), n^a^	247	253
GMT (95% CI)^b^	174.35 (160.62, 189.26)	178.90 (165.45, 193.44)
Day 29, n^c^	247	253
GMT (95% CI)^b^	429.07 (382.77, 480.98)	320.15 (290.55, 352.77)
Model-based GMT (95% CI)	434.42 (397.15, 475.19)	317.55 (290.63, 346.97)
GMR (mRNA-1010 vs active comparator) (95% CI)	1.368 (1.206, 1.552)	
Antibody: influenza B/Austria/1359417/2021 (Victoria lineage) nAb (titer) by MN assay (LLOQ: 14, ULOQ: 10240)		
Baseline (Day 1), n^a^	247	253
GMT (95% CI)^b^	213.06 (185.89, 244.21)	224.95 (191.63, 264.07)
Day 29, n^c^	247	253
GMT (95% CI)^b^	1233.83 (1063.68, 1431.20)	608.53 (523.81, 706.96)
Model-based GMT (95% CI)	1263.26 (1115.89, 1430.08)	600.83 (531.54, 679.14)
GMR (mRNA-1010 vs active comparator) (95% CI)	2.103 (1.766, 2.503)	

The log-transformed antibody levels were analyzed using an ANCOVA model with vaccination group as the fixed variable, log-transformed baseline MN titers as a fixed covariate, adjusting for the randomization stratification factor(s): age group (≥50 to <65 years and ≥65 years) and flu vaccine status in the previous influenza season (received seasonal flu vaccine, did not receive seasonal flu vaccine).

The model-based GMT and GMR and its corresponding 95% CI were obtained by transforming the least square mean estimate and its CI back to the original scale for presentation.

Abbreviations: Ab, antibody; ANCOVA, analysis of covariance; CI, confidence interval; GMR, geometric mean ratio; GMT, geometric mean titer; LLOQ, lower limit of quantification; MN, microneutralization; N, number of participants with nonmissing neutralizing antibody data at the corresponding timepoint; nAb, neutralizing antibody; PPIS, per-protocol Immunogenicity Subset; ULOQ, upper limit of quantification.

^a^Number of participants with nonmissing baseline (day 1) nAb data.

^b^95% CI was calculated based on the t-distribution of the log-transformed values or the difference in the log-transformed values for GM titer and GM fold rise, respectively, and then back-transformed to the original scale for presentation.

^c^Number of participants with nonmissing nAb data at the corresponding visit.

A correlation analysis was performed among participants from both study groups with paired MN and HAI data at days 1 and 29 corresponding to the MN subset of participants. Microneutralization titers were positively correlated with HAI titers, with Pearson correlation coefficients of 0.79 for A/H1N1, 0.74 for A/H3N2, and 0.84 for B/Victoria.

## DISCUSSION

This phase 3 clinical trial demonstrated that a single dose of the trivalent mRNA-1010 vaccine elicited strong humoral immune responses against influenza A and B strains compared with SD influenza vaccine in adults aged ≥50 years. Higher HAI antibody levels with mRNA-1010 compared with the SD influenza vaccine were reflected in the day 29 GMR and SCR differences. These differences were also evident across study subgroups, including among adults aged ≥65 years, participants with high-risk conditions, and those who had received an influenza vaccine in the prior season. Consistent with the HAI findings, MN antibody responses at day 29 were higher in the mRNA-1010 group than in the comparator group for all 3 influenza strains. Superior clinical efficacy of mRNA-1010 relative to SD influenza vaccine has been previously reported, as well as a safety profile consistent with earlier phase 3 evaluations. Overall, these findings support mRNA-1010 as a seasonal influenza vaccine candidate for adults aged ≥50 years.

The observation of higher antibody responses to mRNA-1010 across subgroups, including adults aged ≥65 years, is of interest given the increased risk for severe influenza disease in older populations [3]. Elevated responses in participants with high-risk conditions are also notable, as these groups are typically targeted for enhanced vaccination strategies. In addition, annual influenza vaccination has been associated with attenuated antibody responses in some studies [19, 20]. In this study, we observed that participants in the comparator group who had received an influenza vaccine in the prior season had lower HAI responses across all 3 strains than those who had not received an influenza vaccine in the prior season (Supplementary Table 1). In an exploratory post hoc analysis of a small number of participants (≥50 years) that participated in 2 consecutive mRNA-1010 clinical trials, there was no evidence that year on year mRNA-1010 vaccination blunts efficacy [21].

Hemagglutination inhibition titers are widely used as an immunologic surrogate of protection for influenza vaccines [22, 23], and an HAI titer ≥1:40 has historically been associated with approximately 50% protection against influenza illness in adults [24, 25]. In the present study, one of the immunogenicity endpoints was the frequency of participants with HAI titers ≥1:40 at day 29. We observed that a greater proportion of participants in the mRNA-1010 group achieved HAI titers ≥1:40 at day 29 across all 3 strains compared with the SD influenza vaccine. The lower SCRs observed for the B/Victoria strain can be interpreted in the context of higher baseline antibody levels and a high proportion of participants with preexisting titers ≥1:40, which may limit the magnitude of fold rise responses. Formal analyses of immune correlates were conducted separately in a predefined case-cohort sample and will be reported in a future publication. It is important to note that HAI titers capture only 1 aspect of humoral immunity and do not reflect the full range of immune mechanisms that contribute to protection [26]. Microneutralization responses were positively correlated with HAI titers in the present study, consistent with prior studies showing that HAI and MN assays, while measuring distinct aspects of antibody function, serve as complementary indicators of influenza-specific humoral immunity [27, 28]. In line with the HAI findings, MN antibody responses were higher in the mRNA-1010 group than in the comparator group for all 3 influenza strains. Taken together with the previously reported superior clinical efficacy of mRNA-1010 relative to an SD influenza vaccine [17], these findings support the relevance of HAI as a surrogate immunogenicity marker for influenza vaccines.

Effectiveness of licensed influenza vaccines varies substantially by season and population [5] and may be reduced in older adults, particularly when antigenic match is suboptimal. Improved immune responses have been demonstrated with HD and other enhanced formulations, and randomized controlled trials have shown superior efficacy of HD vaccines compared with SD vaccines supported by higher postvaccination antibody responses [29, 30]. Accordingly, many national immunization technical advisory groups recommend enhanced vaccines for older adults and individuals at increased risk of severe disease. In this context, the present study provides robust, randomized, active-controlled efficacy and immunogenicity data using a relevant comparator. Statistically superior clinical efficacy of mRNA-1010 relative to the SD influenza vaccine was observed in this pivotal phase 3 trial [17], consistent with the immunogenicity differences reported here. In a separate phase 3 trial, mRNA-1010 also elicited higher immune responses than an SD influenza vaccine in adults aged 18–64 years and higher responses than a HD influenza vaccine in adults aged ≥65 years [15], consistent with the trends observed in the present study. Together, findings from both phase 3 studies support the potential of mRNA-1010 as an influenza vaccine candidate that leverages key features of the mRNA platform, including egg-independent production, avoidance of egg-adaptive mutations, flexibility in timing of antigen selection, and the potential for more rapid vaccine updating [31, 32]. By enabling high-fidelity antigen encoding and avoiding egg-based production, these platform characteristics may help preserve antigenic similarity to circulating influenza strains and may contribute to the higher antibody responses observed with mRNA-1010. These features may be particularly relevant in influenza seasons with substantial antigenic drift.

Strengths of this study include the randomized, active-controlled design; the inclusion of a demographically and clinically diverse subset of participants representative of the overall study population, supporting the generalizability of the immunogenicity findings; and the use of complementary immunologic assays, providing internal consistency across surrogate biomarkers. Limitations include relatively small numbers of participants in the immunogenicity subset, especially in certain prespecified subgroups, therefore reducing the precision of subgroup-specific estimates, which may limit generalizability. Clinical studies comparing mRNA-1010 to recombinant influenza vaccines have not been done, resulting in a potential knowledge gap for this type of vaccine. In addition, immunogenicity was assessed through day 29 and against vaccine-matched strains; therefore, the durability of antibody responses beyond day 29 and the breadth of immune responses against antigenically drifted strains were not evaluated in the present analysis and may warrant further study. Finally, HAI assays for this study used cell-derived viruses; because the comparator vaccine was egg-derived, this assay choice could potentially play a role in the observed between-group differences in antibody responses.

In conclusion, this phase 3 study demonstrated that a single dose of mRNA-1010 elicited higher humoral immune responses against influenza A and B strains compared with SD influenza vaccines in adults aged ≥50 years. These immunogenicity findings were consistent across prespecified risk groups, including older adults and participants with high-risk conditions at increased risk for severe influenza. Together with previously reported clinical efficacy and safety data, the results support mRNA-1010 as an enhanced seasonal influenza vaccine candidate for adults aged ≥50 years.

## Supplementary Material

ofag646_Supplementary_Data

## Supplementary Data

Supplementary materials are available at *Open Forum Infectious Diseases* online. Consisting of data provided by the authors to benefit the reader, the posted materials are not copyedited and are the sole responsibility of the authors, so questions or comments should be addressed to the corresponding author.
