# Supplementary material for: Humoral Immunogenicity of mRNA-1010, an mRNA-based Seasonal Influenza Vaccine, in Adults ≥50 Years
Source: Open Forum Infect Dis. 2026 Oct 13;13(10):ofag646. doi: 10.1093/ofid/ofag646 (PMC13693476; doi:10.1093/ofid/ofag646)
Supplement: Supplementary file 1 [file ofag646_supplementary_data.docx]

**Supplementary Material**

Humoral Immunogenicity of mRNA-1010, an mRNA-Based Seasonal Influenza Vaccine, in Adults ≥50 Years

This appendix has been provided by the authors to give readers additional information about the work.

**SUPPLEMENTARY METHODS**

**Trial Design**

This phase 3, randomized, double-blinded, active-controlled, case-driven study was conducted to investigate the safety, efficacy, and immunogenicity of mRNA-1010 versus an active comparator in adults aged ≥50 years. Participants were randomized in a 1:1 ratio to receive a single injection of mRNA-1010 or a licensed inactivated seasonal influenza vaccine, which served as the active comparator. Randomization was stratified by age category (≥50 to <65 years or ≥65 years) at screening and by influenza vaccine status in the previous influenza season (received or not received). Approximately 50% of enrolled participants were to be aged ≥65 years at screening, including approximately 10% who were aged ≥75 years at screening.

Blood samples to support humoral and cellular immunogenicity objectives were collected as follows:

- Participants provided a blood sample on Day 1 (baseline) (prior to study intervention) and on Day 29
- Of these, samples from a pre-specified random subset of approximately 2400 participants from North America—where trivalent influenza vaccine standard-dose comparator was used—were analyzed for hemagglutination inhibition (HAI) titers at Day 1 (baseline) and Day 29 to assess humoral immunogenicity endpoints. Among participants who were randomly selected for HAI testing, an additional randomization was done to select approximately 500 participants (≈250 per group) for the microneutralization (MN) assay to ensure that all participants selected for the MN assay also had HAI measurements

Study intervention preparation, administration, and accountability were performed by designated unblinded study personnel who did not participate in any of the clinical study evaluations. The unblinded study personnel prepared the study intervention out of view of the participant and the blinded study personnel. Unblinded study personnel had no study functions other than the management, documentation, handling, accountability, preparation, and administration of study intervention. They were not involved in participant evaluations or blinded data entry and did not reveal the identity of the individual study intervention assignment to either the participant or the blinded study personnel involved in the conduct of the study.

Unblinded site monitors, who were not involved in other aspects of monitoring, were assigned as the study intervention accountability and treatment confirmation monitors. They had responsibilities to ensure that sites were following all proper study intervention accountability, preparation, and administration procedures. Laboratory personnel responsible for immunogenicity testing of participant samples were blinded to study intervention assignment.

**Study Inclusion Criteria**

Participants were eligible for inclusion in the study only if all of the following criteria applied:

1. Aged ≥50 years at the time of signing the informed consent form
2. Capable of providing signed informed consent, which included compliance with the requirements and restrictions listed in the informed consent form and in the protocol
3. Participants who were assigned female at birth or could become pregnant were eligible to participate if:

- The participant was a person of childbearing potential (POCBP; those who have reached menarche and remain fertile until becoming post-menopausal or permanently sterile) or
- The participant was a POCBP who:
  - Was not breastfeeding/chestfeeding
  - Used an acceptable contraceptive method from ≥28 days before Day 1 to ≥90 days after Day 1
  - Had a negative highly sensitive pregnancy test result at the screening visit and before study intervention

The investigator was responsible for reviewing medical history, menstrual history, and recent sexual activity to minimize the risk of enrolling a participant with an early, undetected pregnancy.

**Study Exclusion Criteria**

Participants were excluded from the study if any of the following criteria applied:

1. Were acutely ill or febrile (temperature ≥38.0°C [100.4°F]) within 72 hours before Day 1
2. Had close contact with someone with laboratory-confirmed influenza infection or with someone who had been treated with antiviral therapies for influenza (e.g., Tamiflu®/oseltamivir; Genentech, Inc., South San Francisco, CA) within 5 days before Day 1
3. Had a history of a diagnosis or condition that, in the judgment of the investigator, was clinically unstable or could have affected participant safety, assessment of study endpoints, assessment of immune response, or adherence to study procedures. *Clinically unstable* was defined as a diagnosis or condition requiring changes in management or medication within 60 days before screening and included ongoing evaluation of an undiagnosed illness that could have resulted in a new diagnosis or condition
4. Reported a history of congenital or acquired immunodeficiency, immunosuppressive condition, asplenia, or recurrent severe infectious disease. HIV-positive participants receiving antiretroviral therapy with a CD4 count ≥500 cells/mm³ and HIV RNA ≤500 copies/mL within the past 365 days were allowed. Certain immune-mediated conditions that were stable and well controlled (e.g., Hashimoto thyroiditis), as well as those that did not require systemic immunosuppressants per Exclusion Criteria 14 and 15 (e.g., asthma, psoriasis, or vitiligo), were allowed at the discretion of the investigator
5. Had dermatologic conditions that could have affected local solicited adverse reaction assessments (e.g., tattoos, psoriasis patches affecting skin over the deltoid injection-site area)
6. Tested positive for influenza by local health authority–approved testing methods within 180 days before Day 1
7. Had a history of anaphylaxis or severe hypersensitivity reaction requiring medical intervention after receipt of any of the following: an mRNA vaccine or therapeutic, components of an mRNA vaccine or therapeutic, influenza vaccine, or components of an influenza vaccine, including egg protein
8. Had a history of coagulopathy or bleeding disorder that was considered a contraindication to intramuscular injection or phlebotomy
9. Had a malignancy within 2 years before Day 1
10. Had a history of Guillain-Barré syndrome after any influenza vaccine
11. Had a history of myocarditis, pericarditis, or myopericarditis—including treatment or any associated symptoms—within 180 days before Day 1
12. Had any medical, psychiatric, or occupational condition—including a reported history of drug or alcohol abuse—that, in the opinion of the investigator, might have posed additional risk due to study participation or could have interfered with the interpretation of study results
13. Underwent a surgical procedure within 7 days before Day 1 or were scheduled to undergo a surgical procedure within 28 days after Day 1. Minor surgical procedures under local anesthesia (e.g., excision of a skin lesion) or diagnostic procedures (e.g., colonoscopy) were allowed
14. Received corticosteroids at ≥10 mg/day of prednisone or equivalent for >14 days in total within 90 days before Day 1 or were anticipating the need for corticosteroids at any time during the study

Epidural or major joint intra-articular injections (e.g., knee, hip, or shoulder) or other injections with long-acting corticosteroids within 28 days before or anticipated within 28 days after Day 1 were not allowed. Inhaled, nasal, and topical steroids were allowed

15. Had received systemic immunosuppressive treatment, including long-acting biological therapies that affect immune responses (e.g., infliximab), within 180 days before Day 1 or planned to do so during the study
16. Had been treated with antiviral therapies for influenza (e.g., Tamiflu®) within 180 days before Day 1
17. Had received systemic immunoglobulins or blood products within 90 days before Day 1 or planned to do so during the study
18. Had received any vaccine authorized or approved by a local health agency within 28 days before Day 1 or planned to do so within 14 days after Day 1
19. Had received a licensed seasonal influenza vaccine within 180 days before Day 1 or planned to do so (outside of this study) at any time during the study
20. Had received an investigational seasonal influenza vaccine within 1 year before Day 1. Participants from mRNA-1010-P304 Season 1 were not eligible to reenroll in Season 2
21. Were unsure whether they had received a licensed or investigational seasonal influenza vaccine within 1 year before Day 1
22. Had donated ≥450 mL of blood products within 28 days before Day 1 or planned to donate ≥450 mL within 28 days after Day 1
23. Had participated in a clinical study with an investigational treatment within 90 days before Day 1 (baseline), based on medical history, or planned to do so while participating in this study. Participants could continue in prior interventional study follow-up activities, provided that this did not involve further investigational treatment or procedures that might have affected safety or study endpoints and that participation did not result in a protocol breach. Interventions such as counseling, biofeedback, and cognitive therapy were allowed
24. Were working or had worked as study personnel or were immediate family or household members of study personnel, study staff, or sponsor personnel

**Statistical Analysis**

**Study Analysis Sets**

| **Analysis Set** | **Description** |
| --- | --- |
| Full analysis set (FAS) | All randomized participants who received any study vaccination. Participants will be analyzed according to the treatment group to which they are randomized |
| Immunogenicity subset | A pre-specified subset of participants in the FAS and have Day 1 (baseline) and Day 29 humoral immunogenicity samples. Participants will be analyzed according to the study vaccination group to which they are randomized |
| Per-protocol immunogenicity subset (PPIS) | All participants in the immunogenicity subset who received the planned dose of study vaccination and had no important protocol deviations that affected the immunogenicity assessment  Participants with reverse transcriptase–polymerase chain reaction–confirmed influenza infection between Day 1 (baseline) and Day 29 will be removed from the PPIS. The PPIS will be decided blindly by the blinded study team before the database lock for the interim or final analysis |

PPIS size was determined based on the similarity to powered subsets from previous immunogenicity studies.

**Immunogenicity Analyses**

The following 3 strains will be measured by HAI assay:

- Influenza A/H1N1/Wisconsin/67/2022
- Influenza A/H3N2/Massachusetts/18/2022
- Influenza B/Victoria/Connecticut/01/2021

Analysis of secondary immunogenicity endpoints was conducted by measuring geometric mean titers (GMTs) on Day 29, as measured by HAI. An analysis of covariance (ANCOVA) model was carried out. The model included the log-transformed HAI titers on Day 29 as the dependent variable, treatment group as the fixed variable, and log-transformed baseline HAI titers as a fixed covariate, adjusted for the stratification factors. The geometric least-squares mean (GLSM) and corresponding 95% CIs in log-transformed scale, estimated from the model, were back-transformed to obtain estimates in the original scale as an estimate of the GMT.

The geometric mean ratio (GMR) for mRNA-1010 versus active comparator was estimated by the ratio of GLSM and the corresponding 2-sided 95% CI, which was provided to assess the treatment difference and was calculated by back-transforming the difference estimated from the ANCOVA model. The corresponding 2-sided 95% CI of GMR was provided to compare the difference in immune response between the mRNA-1010 group and the active comparator group on Day 29. Immunogenicity analyses were descriptive, and no formal hypothesis testing was planned. For interpretation of the GMR, a lower bound of the 95% CI >0.667, which is 1/1.5, was used, corresponding to the reciprocal of the FDA-recommended criterion that the upper bound of the 2-sided 95% CI for GMT _U.S. licensed vaccine_/GMT _investigational vaccine_ should not exceed 1.5.

Descriptive statistics (n, median, min, max) were provided for the GMT of HAI titers with corresponding 95% CI at each time point. The 95% CIs were calculated based on the t-distribution of the log-transformed values and then back-transformed to the original scale for presentation. In addition, the GMT with corresponding 95% CI were plotted in bar charts at each time point.

The geometric mean fold rise (GMFR) comparing Day 29 to Day 1 (baseline) as measured by HAI were analyzed using the same descriptive statistics, and the GMT analysis was provided for GMFR as measured by HAI with corresponding 95% CI on Day 29 over Day 1 (baseline).

The number and percentage of participants with seroconversion on Day 29 or an HAI titer ≥1:40 on Day 29 were provided with a 2-sided 95% CI using the Clopper-Pearson method. To compare the seroconversion rates between the treatment groups, the Miettinen-Nurminen method was used to calculate the 95% CI for the difference in seroconversion rates on Day 29. The number and percentage of participants with HAI titers ≥1:40 on Day 29 was provided with 2-sided 95% CIs using the Clopper-Pearson method.

The analysis of exploratory immunogenicity endpoints included the GMT on Day 29 as measured by MN assay and the GMFR comparing Day 29 to Day 1 (baseline), as measured by MN assay. The same ANCOVA model and descriptive statistics were used for the secondary analysis and was carried out for the GMT on Day 29, as measured by MN assay.

**Assays**

Humoral immune responses were assessed using validated HAI and MN assays. HAI assays were performed at IQVIA Laboratories, and the MN assays were performed at VisMederi. All sample testing was conducted in a blinded manner, with laboratory personnel performing the assays blinded to vaccination group.

The HAI assay was performed using guinea pig red blood cells and cell-propagated influenza viruses, in accordance with World Health Organization (WHO) guidelines. Prior to testing, serum samples were treated with receptor-destroying enzyme (RDE II “Seiken”; Accurate Chemical, Cat. No. YCC340-122) to remove nonspecific inhibitors. Three volumes of RDE were added to 1 volume of serum, followed by incubation for 18 ± 2 hours at 37 ± 2°C. Residual RDE was inactivated by heating at 56 ± 2°C for 30 ± 5 minutes, which also heat-inactivated the samples. Physiological saline was then added to achieve a final 1:10 dilution. RDE-treated sera were subsequently adsorbed with 10% guinea pig red blood cells in Dulbecco’s phosphate-buffered saline at a 3:5 red blood cell suspension-to-serum ratio. Samples were incubated at 2–8°C for 1 hour with intermittent mixing and then centrifuged at approximately 1700 × g for 10 minutes. A second adsorption was performed if nonspecific agglutination remained. Serum samples were tested using a standardized viral input of 4 hemagglutination units and 2-fold serial dilutions, with a starting dilution of 1:10 and a maximum dilution of 1:10,240. HAI titers were defined as the reciprocal of the highest serum dilution that inhibited hemagglutination, as evidenced by the formation of a distinct red blood cell halo. Assay performance characteristics—including precision, relative accuracy, and limits of quantification—were established during validation using incurred study samples meeting pre-defined acceptance criteria.

The MN assay was conducted using WHO-recommended strains for the 2024–2025 Northern Hemisphere influenza season (A/H1N1/Wisconsin/67/2022, A/H3N2/Massachusetts/18/2022, and B/Austria/1359417/2021 (Victoria lineage), which were antigenically matched to the vaccine components evaluated in this study. Serum samples were heat-inactivated but were not treated with RDE. The starting dilution was 1:10, with a maximum dilution of 1:10,240. A single-cycle MN assay was performed in Madin-Darby canine kidney cells using cell-derived viruses. Neutralizing activity was quantified by enzyme-linked immunosorbent assay targeting viral nucleoprotein. Neutralization titers (NT50) were calculated using a continuous method relative to virus-positive and cell-only control wells.

**Tables**

**Supplementary Table 1. Subgroup Analysis of HAI Antibody Levels and Seroconversion for Vaccine-Matched Seasonal Influenza A and B Strains Measured by HAI Assay on Day 29 by Influenza Vaccination in the Previous Influenza Season (Per-Protocol Immunogenicity Subset)**

|  | mRNA-1010 37.5 ug | Standard-Dose Licensed Influenza Vaccine |
| --- | --- | --- |
| **Influenza vaccination in the previous influenza season: received** | | |
| *Influenza A H1N1 Wisconsin/67/2022* | | |
| Day 29 |  |  |
| n | 594 | 596 |
| Model-based GMT (95% CI) | 117.48 (110.64–124.73) | 60.54 (57.02–64.27) |
| GMR (95% CI) | 1.941 (1.783–2.112) |  |
| Seroconversion, n (%),^a^ (95% CI^b^) | 276 (46.5) (42.40–50.57) | 87 (14.6) (11.86–17.69) |
| *Influenza A H3N2 Massachusetts/18/2022* | | |
| Day 29 |  |  |
| n | 594 | 596 |
| Model-based GMT (95% CI) | 129.12 (121.05–137.73) | 80.95 (75.90–86.34) |
| GMR (95% CI) | 1.595 (1.456–1.747) |  |
| Seroconversion, n (%),^a^ (95% CI^b^) | 314 (52.9) (48.76–56.94) | 193 (32.4) (28.64–36.30) |
| *Influenza B/Victoria/Connecticut/01/2021* | | |
| Day 29 |  |  |
| n | 594 | 596 |
| Model-based GMT (95% CI) | 228.20 (217.10–239.88) | 131.31 (124.93–138.02) |
| GMR (95% CI) | 1.738 (1.620–1.865) |  |
| Seroconversion, n (%),^a^ (95% CI^b^) | 222 (37.4) (33.47–41.40) | 54 (9.1) (6.88–11.66) |
| **Influenza vaccination in the previous influenza season: not received** | | |
| *Influenza A H1N1 Wisconsin/67/2022* | | |
| Day 29 |  |  |
| n | 573 | 579 |
| Model-based GMT (95% CI) | 184.09 (168.35–201.30) | 106.18 (97.15–116.06) |
| GMR (95% CI) | 1.734 (1.528–1.967) |  |
| Seroconversion, n (%),^a^ (95% CI^b^) | 366 (63.9) (59.79–67.81) | 231 (39.9) (35.88–44.01) |
| *Influenza A H3N2 Massachusetts/18/2022* | | |
| Day 29 |  |  |
| n | 573 | 579 |
| Model-based GMT (95% CI) | 171.29 (157.74–186.01) | 107.99 (99.49–117.22) |
| GMR (95% CI) | 1.586 (1.412–1.782) |  |
| Seroconversion, n (%),^a^ (95% CI^b^) | 396 (69.1) (65.15–72.87) | 287 (49.6) (45.42–53.72) |
| *Influenza B/Victoria/Connecticut/01/2021* | | |
| Day 29 |  |  |
| n | 573 | 579 |
| Model-based GMT (95% CI) | 277.07 (258.22–297.30) | 171.24 (159.64–183.67) |
| GMR (95% CI) | 1.618 (1.465–1.787) |  |
| Seroconversion, n (%),^a^ (95% CI^b^) | 304 (53.1) (48.87–57.20) | 178 (30.7) (27.00–34.68) |

Antibody values reported as below the LLOQ are replaced by 0.5 × LLOQ. Values greater than the ULOQ are converted to the ULOQ. The log-transformed antibody levels were analyzed using an analysis of covariance model with vaccination group as the fixed variable and log-transformed baseline HAI titers as a fixed covariate, adjusted for the randomization stratification factor age group (≥50 to <65 years and ≥65 years). The model-based GMT and GMR, and their corresponding 95% CIs, were obtained by transforming the least-squares mean estimates and their CIs back to the original scale for presentation.

Abbreviations: GLSM, geometric least-squares mean; GMR, geometric mean ratio; GMT, geometric mean titer; HAI, hemagglutination inhibition; LLOQ, lower limit of quantification; mRNA, messenger RNA; n, the number of participants with non-missing HAI antibody data at the corresponding time point; ULOQ, upper limit of quantification.

^a^Rate of seroconversion is defined as the proportion of participants with either a baseline HAI titer <1:10 and a

post-baseline titer ≥1:40 or a baseline HAI titer ≥1:10 and a minimum 4-fold rise in post-baseline HAI antibody titer.

^b^95% CIs were calculated using the Clopper-Pearson method.
